# ORCID coverage in research institutions—Readiness for partially automated research reporting

**DOI:** 10.3389/frma.2022.1010504

**Published:** 2022-11-10

**Authors:** Kathrin Schnieders, Sandra Mierz, Sabine Boccalini, Wibke Meyer zu Westerhausen, Christian Hauschke, Stephanie Hagemann-Wilholt, Sonja Schulze

**Affiliations:** ^1^Centralized Reporting, Osnabrueck University, Osnabrueck, Germany; ^2^Open Science Lab, Leibniz Information Centre for Science and Technology - TIB, Hannover, Germany; ^3^University Library, Osnabrueck University, Osnabrueck, Germany; ^4^PID Competence Centre, Leibniz Information Centre for Science and Technology - TIB, Hannover, Germany

**Keywords:** (semi-)automated research reporting, linked open metadata, current research information system (CRIS), persistent identifier (PID), ORCID, ROR, FREYA, OpenAlex

## Abstract

Reporting and presentation of research activities and outcome for research institutions in official, normative standards are more and more important and are the basis to comply with reporting duties. Institutional Current Research Information Systems (CRIS) serve as important databases or data sources for external and internal reporting, which should ideally be connected with interfaces to the operational systems for automated loading routines to extract relevant research information. This investigation evaluates whether (semi-) automated reporting using open, public research information collected *via* persistent identifiers (PIDs) for organizations (ROR), persons (ORCID), and research outputs (DOI) can reduce effort of reporting. For this purpose, internally maintained lists of persons to whom an ORCID record could be assigned (internal ORCID person lists) of two different German research institutions—Osnabrück University (UOS) and the non-university research institution TIB—Leibniz Information Center for Science and Technology Hannover—are used to investigate ORCID coverage in external open data sources like FREYA PID Graph (developed by DataCite), OpenAlex and ORCID itself. Additionally, for UOS a detailed analysis of discipline specific ORCID coverage is conducted. Substantial differences can be found for ORCID coverage between both institutions and for each institution regarding the various external data sources. A more detailed analysis of ORCID distribution by discipline for UOS reveals disparities by research area—internally and in external data sources. Recommendations for future actions can be derived from our results: Although the current level of coverage of researcher IDs which could automatically be mapped is still not sufficient to use persistent identifier-based extraction for standard (automated) reporting, it can already be a valuable input for institutional CRIS.

## Introduction

The growing amount of third party funding in German research institutions and the increasing complexity of funding allocation has produced a need for more precise monitoring (CRIS) and evaluation (reporting) of research performance and documentation.^1^ Internal reporting purposes and recipients have to be addressed in different reporting cycles (*ad-hoc*, annual, quarterly, and monthly reporting), but external stakeholders—funders, politics, and the public—also need to be informed about research performance. These requirements have fostered the development and differentiation of comprehensive reporting systems as quality management and control instruments for the governance of research institutions (Wissenschaftsrat, 2011; Internationale Expertenkommission zur Evaluation der Exzellenzinitiative, 2016). CRIS are designed to create a consistent, quality-assured, and complete database to meet these information requirements for a wide range of stakeholders (Biesenbender et al., 2019). Complex requirements challenge research institutions in many ways: In the cumbersome collection of data, their great heterogeneity, restricted availability, and varying quality. In many cases, the data have to be created with much effort in the first place or gathered (manually) from different sources. Common reporting and metadata standards have to be defined and accepted across institutional boundaries in order to establish comparability at all (Hicks et al., 2015; Biesenbender and Herwig, 2019). With the development of the Core Data Set on Research Activities (KDSF—Kerndatensatz Forschung), the German Council of Science and Humanities has initiated an important process for standardization of research reporting which helps research institutions to set up an institutional research reporting system with comparable data (Wissenschaftsrat, 2016).

A promising approach to fulfill reporting requirements is to connect and integrate data from external repositories and databases, which provide open data with free licenses and open PIDs into local CRIS. This pragmatic approach allows a central collection of data on research activities and output of individual researchers for reporting purposes, enrich researcher profiles, and follows the principle of “enter once, reuse often”.^2^

PIDs are unique strings that allow the unambiguous and sustainable identification, referencing capability and linking of research resources, researchers and associated institutions. They are usually accompanied by metadata that describe referenced objects more or less extensively, for example the context of an object, person, or institution. They support discovery and unambiguous identification of research organizations (ROR ID),^3^ researchers (ORCID),^4^ and their published research results (DOI)^5^ and encourage the adoption of interoperable metadata standards. While proprietary service providers often do not make their metadata comprehensively accessible, community driven services such as DataCite,^6^ ORCID or ROR have been established in the science system and provide metadata *via* open interfaces. Thus, they are among the key players for implementing Open Science practices.^7^

While the use of open organizational identifiers like ROR ID, introduced in 2019, is still relatively new,^8^ PID service infrastructure providers such as DOI registry agencies DataCite and Crossref,^9^ already support the specification of organizational IDs in their metadata schemes and therefore contribute significantly to their dissemination. Since its beginning in 2012, ORCID has become increasingly popular and has recorded a steady growth in ORCID registrations (Bertelmann et al., 2015, 2019). The scope of PIDs is under constant development. For example, DOIs metadata sets are defined for software, projects, instruments, events, and other entities.^10^ Research entities can be linked to each other *via* PIDs and these links can be visualized, e.g., *via* graphs.^11^ These developments support an increasingly fine-grained mapping of scientific activities and outputs (Lavasa et al., 2018; Meadows et al., 2019).

If PIDs were used consistently and were integrated in all information infrastructure systems it should be possible to start from a ROR ID to receive an almost complete set of DOIs, representing the research output of the organization under investigation. This leads to the question, whether (semi-) automated extraction of external research data using PIDs can already be leveraged to extend and maintain content in CRIS.

As research outputs are usually associated with authors rather than organizations, an intermediate step might be useful to determine the people belonging to an organization, and subsequently the research output of each person. The query process is accordingly two-stage: first the relation organization to people and then the relation person to works.

This paper will solely focus on the first stage of the query—the connection between an organization and its affiliated researchers—and on the question to what extent an unambiguous mapping can be carried out automatically.

Is the coverage and quality of metadata from open data sources sufficient to identify researchers of a specific institution? To what extent can ORCID iDs automatically be mapped to researchers belonging to organizations by ROR ID? Is there a differential prevalence of ORCID registrations by type of organization, research area or discipline?

## Related works

More general studies of coverage in open data sources are not yet available in large but in considerable numbers. Of these studies, Visser et al. (2021) in particular is strongly received and detects differences in document coverage between Scopus, Web of Science, Dimensions, Crossref, and Microsoft Academic. Chudlarský and Dvorák (2020) analyze the suitability of open data sources for research evaluation from the perspective of using open citation information in contrast to commonly used proprietary citation databases.

A new study by van Eck and Waltman (2022) investigates Crossref as a bibliographic data source and gives an overview about the availability of reference lists, abstracts, ORCID iDs, author affiliations, funding information, and license information in the metadata provided by Crossref. They find that the ORCID coverage for journal articles in Crossref increased from 0 to 39% between 2012 and 2022.

Haak et al. (2018) describe the motivation of researchers and commercial vendors to use open PIDs and investigate under which conditions ORCID iD and DOI can be used in research evaluation. Lauridsen and Melchiorsen (2020) analyze how complete publication lists using ORCID query of the Web of Science API are compared to more involved intellectual searches. They conclude, that researchers' motivation to maintain their ORCID record up to date is crucial for automated reporting and evaluation.

Mugabushaka et al. (2022) outline that there is only limited coverage and quality of funding data in infrastructures like Crossref, Web of Science, and Scopus.

Mendes Moreira et al. (2015) describe, how CRIS can synchronize their data based on ORCID, focusing on consistency management. Baglioni et al. (2021) derive five misapplications of ORCID iDs in the ORCID registry, which are considered but do not affect our subsequent study.

Albert et al. (2021) investigate ORCID coverage in context of developing an identity-driven authorship prediction and reveal that only 6% of Weill Cornell papers of all times in PubMed have an ORCID iD asserted. This percentage raised to a level of 22% for publication year 2019.

One of the most detailed and up to date overviews of ORCID coverage is provided by Porter (2021): It shows a comparison of ORCID coverage by country and research area. Portugal seems to be front runner (67%), whereas Germany is mid-range (~33%). ORCID adoption by research area reveals a wide bandwidth spreading from Earth Sciences (45%) to Law and Legal Studies (~25%).

In their exploration of ORCID's potential as a research data source for meta research in the field of science and research dynamics Costas et al. (2022) examine the coverage of specific entry types (works, employment, funding, other identifiers, etc.) in ORCID records.

Priem et al. (2022) briefly outline how OpenAlex achieves ROR coverage of ~94% for institutions. They state that about the half of over 209 million works contain DOIs. Neylon and Kramer (2022) dig deeper into OpenAlex metadata such as ORCID or DOI to assess the added value of OpenAlex compared to Crossref data.

## Materials and methods

### Study design

The methodological approach is based on the following analysis steps for answering the research questions:

- Identification of internal ORCID coverage for two research institutions.
- Selection of open public data sources.
- Comparison of internal lists with data compiled by public data sources.
- Detailed analysis of discipline specific ORCID coverage for UOS.

The general objective of the study is to explore the status quo and the development potentials of PIDs for two different types of research institutions (university vs. a non-university) in terms of ORCID iDs usage and coverage. UOS and TIB are chosen as objects of investigation. UOS is a German mid-sized university with about 14,000 students and 1,104 academic researchers (data December, 2021). TIB serves as the German National Library of Science and Technology and is a member of the Leibniz Association of non-university research institutions. Its research focus is on information infrastructures. In addition, TIB is engaged with the DOI registration agency DataCite and is the administrative lead of the TIB DOI consortium^12^ and the ORCID DE consortium.^13^ TIB has about 550 employees, of which ~80 are academic staff, and 4 of them professors (data June, 2021).

The investigation is limited to researchers employed at the research institutions in June 2021 (creation of internal ORCID person lists). For UOS only persons with status full professor or project manager in third-party^14^ funded projects are considered.

### Identification of internal ORCID coverage for UOS and TIB

Initially, as reference for the comparison and assessment of ORCID coverage in external data sources, the internal ORCID coverage in institutional systems of UOS and TIB is calculated.

Therefore, lists of researchers' full names enriched with registered ORCID iDs are compiled and manually verified by both institutions. The internal list of UOS contains additional information about the researchers' field and research topic retrieved by local CRIS.

The total size of the internal person lists include 93 researchers for TIB and a total sample size of 264 researchers for UOS. The discrepancy with the number of academic staff at TIB is due to the fact that non-academic staff also publish at TIB, for example in journals of applied library and information science.

### Selection of open data sources

The initial selection of data sources is based on a set of minimal requirements to ensure reusability for a (semi-) automated reporting. The data

- are relevant in context of research reporting (relevance),
- can be queried by an API (automation),
- are provided under an open free license (reusability and availability),
- are referenced by a PID (disambiguation).

#### FREYA PID graph

Generally speaking, a PID graph comprises a structure of entities, each identified by a PID, and their connections. The FREYA PID graph was developed by DataCite in an EC-funded project and launched in May 2020.^15^ It aims at improving discovery, navigation, retrieval, and access of research resources by combining scholarly resources. Organizations are identified by their ROR or Grid ID, people by their ORCID iD, publications by their DOI and funders by their CrossRef Funder ID.^16^ For a connection to be established between two entities one of the objects needs to reference the other objects PID within their metadata. Querying FREYA PID graph for an entity's associated metadata and connected entities is possible *via* a GraphQL API.^17^ The query (organization-people) is published under a BSD (Berkeley Software Distribution) license and may be reused (Mierz, 2022).

#### ORCID

ORCID was launched in 2012 with the goal to provide an open community driven PID for researchers so they are “uniquely identified and connected to their contributions across disciplines, borders, and time.”^18^ Additionally, researchers and dedicated trusted parties have the opportunity to populate the ORCID record with metadata describing professional activities:^19^ affiliations to organizations, grants and publications reference their PIDs, thus creating a PID graph.

The ORCID public API can be queried using either the Grid ID, Ringgold ID or ROR ID (or preferably) their combination to retrieve people affiliated with an organization.

Because the connection between organization and people *via* their affiliation as defined by the ORCID public API is quite broad, the detailed ORCID records referring one of the organization's IDs are retrieved and filtered to distinguish between persons currently employed (end-date of employment is empty) and former employees or students. The chain of queries and further documentation is published for reuse (Fenner et al., 2011; Mierz, 2022).

#### OpenAlex

At the beginning of 2022, the non-profit organization OurResearch launched a new open data source named OpenAlex. It intends to be a replacement for the Microsoft Academic Graph which was discontinued at the end of 2021 (Singh Chawla, 2022).

OpenAlex is centered around five research entity types and their connections: works, authors, venues, institutions, and concepts. While it is not explicitly marketed as a PID graph, each of the entities is identified by at least one PID and their connections are also established *via* PIDs, effectively making it a graph. OpenAlex aggregates data of various open sources namely Crossref, ROR, ORCID, Unpaywall among others, standardizes and normalizes it. The enriched data are distributed under CC0 license (Priem et al., 2022).

Querying OpenAlex for an entity's associated metadata and connected entities is possible *via* REST API.^20^ Each entity type has its own endpoint with specific filters and retrieval options. We use the authors endpoint to filter all affiliated authors based on their last_known_institution.ror value. The query (organization-people) is published for further use (Mierz, 2022).

### Comparison of data from public data sources with internal person lists

In August 2021, an initial comparison of internal ORCID person lists and external queried ORCID person lists *via* FREYA PID graph was conducted, repeated and extended to ORCID itself and OpenAlex in March 2022 (Schnieders and Mierz, 2021). As stated before all data sources are requested using an organization-people query, to evaluate how many researchers can be uniquely and automatically assigned to their research institutions *via* ROR ID.

The results from querying FREYA PID graph and OpenAlex include former employees and students of the institutions, whereas the ORCID query is designed to retrieve only current affiliated employees, as these are relevant for reporting purposes. The results for each data source (researchers' full names and ORCID iDs) are mapped against the internal person lists of both institutions.^21^ The percentage of matches is determined as measure for ORCID coverage.

### Detailed analysis of discipline specific ORCID coverage for UOS

Differences in research discipline specific coverage are examined using the example of UOS. The UOS is a German mid-sized university, which offers a wide range of subjects and research disciplines in “the areas of Humanities, Social Sciences, Natural Sciences, Law and Economics”.^22^

As the internal list of UOS contains additional information about the researchers' fields and research topics retrieved by local CRIS the query results can be analyzed by scientific research area.

## Results

Table 1 shows values of ORCID coverage and the relationship of verified ORCID iDs to researchers in total for both institutions in selected open data sources.

**Table 1:** ORCID coverage for selected open data sources for UOS and TIB.

			**Open data sources**			
**Institution**	**Internal data source**		**FREYA 2021**	**FREYA 2022**	**ORCID**	**OpenAlex**
TIB	Researchers in total	93	35 (38%)	38 (41%)	25 (27%)	12 (13%)
	Verified ORCID iDs	83 (89%)	35 (42%)	38 (46%)	25 (30%)	12 (14%)
UOS	Researchers in total	264	49 (19%)	52 (20%)	43 (16%)	67 (25%)
	Verified ORCID iDs	108 (41%)	49 (45%)	52 (48%)	43 (40%)	67 (62%)

### Internal coverage

Based on the internal ORCID person list, for about 89% of TIB researchers (respectively, 41% of UOS researchers) an ORCID iD can be verified. This subset of verified ORCID iDs and the associated internal coverage limits external ORCID coverage, that can be achieved by querying external data sources and verifying the retrieved ORCID iDs against the internal ORCID person list.

### External ORCID coverage in selected data sources

#### FREYA PID graph

Based on the set of results from August 2021, with a total of 77 persons affiliated to TIB *via* ROR ID, the data show an overlap for 35 researchers, representing 38% of all TIB researchers and 42% of those with verified ORCID iDs.

The identically conducted comparison in March 2022 shows that of the entire query result with a total of 90 entries, 38 persons can clearly be mapped with the internal ORCID person list, increasing coverage to 41% of all TIB researchers and 46% of verified ORCID iDs, respectively.

For UOS in August 2021 a total of 473 affiliated ORCID iDs is found *via* FREYA PID graph. In March 2022 the query result contains 528 entries. Mapped to the internal ORCID person list, ORCID coverage for UOS in FREYA PID graph raises from 45% (49 researchers) to 48% (52 researchers) for verified ORCID iDs in this time period. The coverage based on all UOS researchers (full professors and project managers) raises from 19 to 20%.

#### ORCID

The query result *via* ORCID public API (focusing on current employees), with a total result of 59 entries, gives an intersection of 25 researchers with the internal ORCID person list of TIB, representing 27% of TIB researchers in total and 30% of those with verified ORCID iDs.

For UOS 43 researchers of 157 affiliated ORCID iDs can be mapped to the internal list *via* affiliation information in their public ORCID records, 16% of UOS researchers in total but 40% of those with verified ORCID iDs.

#### OpenAlex

Querying the OpenAlex graph *via* ROR ID for TIB researchers produces a result set of 15 persons. Matched with the verified ORCID iDs of the internal TIB list, 12 persons can be identified unambiguously, which means 13% coverage or 14% for verified ORCID iDs. A comparison of the internal list of UOS with external queried person list (with 383 entries) shows a coverage of 62% for verified ORCID iDs and 25% for researchers in total (67 researchers).

### Discipline specific ORCID coverage for UOS

#### Internal ORCID coverage

Table 2 presents the distribution of the 108 verified ORCID iDs over faculties and departments for researchers at UOS.

**Table 2:** Internal ORCID distribution by faculties and departments for UOS.

**Faculties and departments represented in local CRIS**	**Researchers in total**	**Verified ORCID iDs**	**Coverage in %**
Cultural studies and social sciences	38	10	26%
Educational and cultural studies	44	6	14%
Physics	14	12	86%
Biology and chemistry	39	26	67%
Mathematics and computer science	31	13	42%
Language and literary studies	23	4	17%
Human sciences	31	20	65%
Business administration and economics	17	7	41%
Law	19	5	26%
Administration incl. library	8	5	63%
In total	264	108	41%

Discipline specific distribution patterns of internal ORCID coverage range from 86% (Physics) to 14% (Educational and Cultural Studies). Researchers in disciplines related to the Natural Sciences and (Information) Technology (STEM) appear to use ORCID iDs already to a higher extend than scholars in Social Sciences and Humanities (SSH).

#### External ORCID coverage by data sources

**Figure 1:**
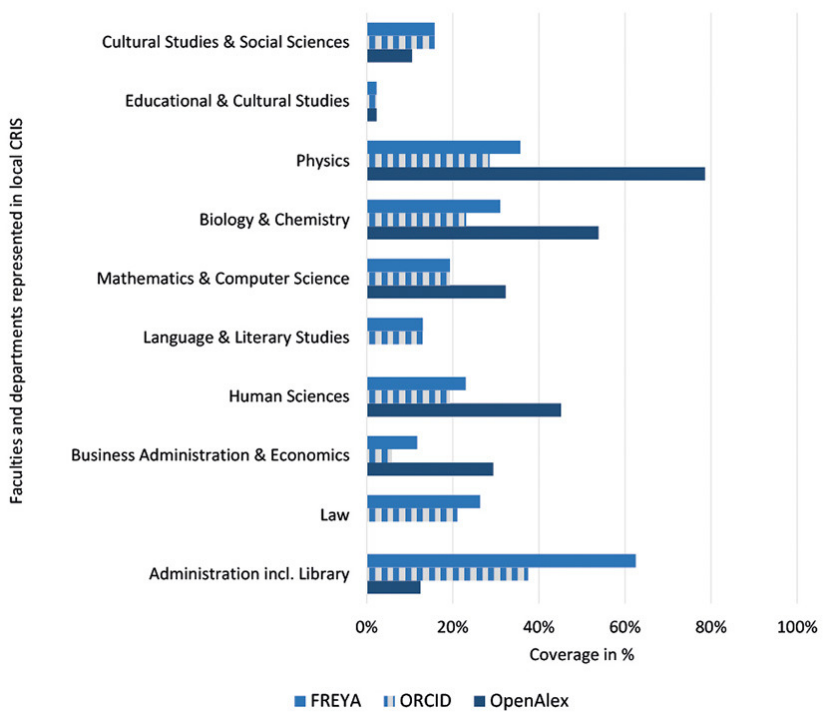
ORCID coverage for researchers (UOS) in total in external data sources.

**Figure 2:**
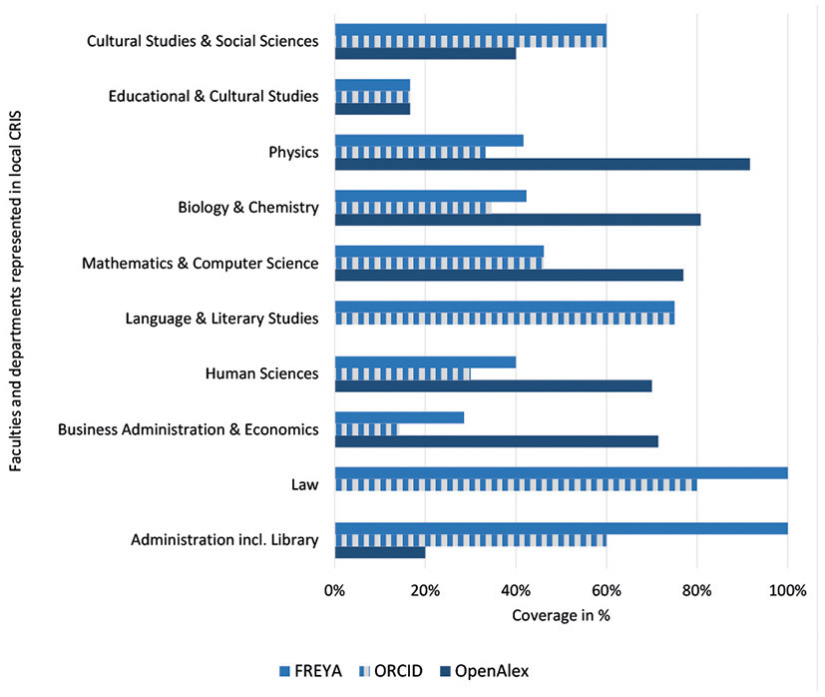
ORCID coverage for verified ORCID iDs (UOS) in external data sources.

Figure 1 illustrates the discipline specific ORCID coverage in external data sources for UOS researchers in total in March 2022, while Figure 2 is focused on the subset with verified ORCID iDs.

##### FREYA PID graph

As can be seen in Figure 1 ORCID coverage in FREYA PID graph for the majority of faculties and departments ranges between 12 and 28%. Outliers form the School of Education and Cultural Studies (2%), the School of Physics (36%), and the Administration including University Library (63%).

Focusing on the subset with verified ORCID iDs in Figure 2 this picture is changing, leading to a different ranking of disciplines: In this subset the School of Law (100% coverage), employees in Administration and Library (100%), and the School of Linguistics and Literary Studies (75%) seem to be covered quite good by FREYA whereas the Schools of Education and Cultural Studies and Business Administration and Economics achieve a coverage of <30%.

##### ORCID

As described in the methodological part, FREYA is harvesting ORCID so affiliation information in public ORCID records should be integrated in FREYA. In most cases, the coverage rates in ORCID are quite similar to those in FREYA. Nevertheless, some cases show differences, for example for the School of Business Administration and Economics.

##### OpenAlex

Figure 1 shows that coverage data for OpenAlex are spread over a wider range: Researchers in Law and Language and Literature Studies are not found at all *via* ROR query, whereas the School of Physics achieves satisfying values (79%).

The subset of verified ORCID iDs in Figure 2 has a bandwidth of 0% (Schools of Law and Language and Literature Studies) to 92% (School of Physics). The pattern of distribution however is similar to coverage data shown in Figure 1 so the ranking of disciplines is preserved.

## Discussion

### Internal ORCID coverage

The high internal ORCID coverage of TIB (89%) compared to UOS (41%) is striking. However, assuming ~33% of German researchers actively using ORCID between 2015 and 2019 (Porter, 2021), UOS also performs well. TIB in contrast is a non-university research institution with special focus: PIDs and information infrastructures are its central research subjects and daily business, e.g., TIB is the leader of the German ORCID consortium. Therefore, a higher affinity for using PIDs among TIB's research staff can be assumed.

### External ORCID coverage

Table 1 shows that ORCID coverage differs between external data sources and institutions. While TIB has its highest coverage in FREYA PID graph (46%), UOS performs best in OpenAlex (67%). The limiting factor for querying coverage in all external data sources is of course the internal coverage of verified ORCID iDs.

Despite a higher internal coverage, it is remarkable that TIB does not perform better in ORCID than UOS. Since the ORCID public API query uses institutional PIDs in employment entries with visibility set to *public* by researchers (Mierz, 2022), TIB could achieve better external coverage in ORCID through consistent maintenance of affiliation information in ORCID records, given its high internal coverage. This applies also for UOS of course: Strengthening the usage of ORCID at UOS (improving internal coverage) would only positively impact external coverage if maintenance and visibility of affiliation information in ORCID records is also addressed.

The reason for the discrepancies between TIB's and UOS' performance in FREYA and OpenAlex is probably due to the different data sources of both systems. While FREYA relies on DataCite Event Data and has its focus on research data and software,^23^ OpenAlex uses Crossref metadata as source of works and has currently no implementation of DataCite's metadata schema so far (Priem et al., 2022). As already described in the methodological part, FREYA fully integrates public data of ORCID records. OpenAlex on the other hand uses ORCID records to enrich its author profiles with name variants. Only ORCID iDs linked with works in Crossref metadata are included (usually publisher's publications) (Priem et al., 2022).

Considering the high coverage of UOS in OpenAlex one has to bear in mind, that only full professors and project managers are included in the internal list. These researchers usually have an extensive publication list. For other status groups of persons (early career researchers, research assistants, i.e.), different coverage rates might result.

FREYA as well as OpenAlex uses metadata of research outputs while ORCID's mapping of research organizations and affiliated researchers is independent from publications. This requires researchers' engagement in maintaining their public ORCID records and the proactive use of their ORCID iDs in publication processes (Lauridsen and Melchiorsen, 2020).

### Discipline specific ORCID coverage

Table 2 shows ORCID coverage of UOS researchers by faculties and departments. The size of the faculties' representation in the internal list depends on two factors: First, the number of disciplines that form a faculty and their number of chairs (full professors). Second, the number of third-party funded projects acquired (project managers). As UOS is a mid-sized university, numbers are at a low level when distribution over faculties is considered. Especially due to the study design, where the internal list is as limited as described. More than 100 additional ORCID iDs of current employees are found with the ORCID query, but not further analyzed as they are not part of the internal list. Given the small numbers in the results, conclusions should be drawn with care. Nevertheless, some tendencies can be observed parallel to Porter (2021): ORCID is more prevalent in STEM disciplines compared to SSH. The figures for Administration and Library are misleading, as only a small part of their employees are included in the internal list. These people are often engaged in projects that are directly related to PIDs.

ORCID coverage in external data sources for researchers in total shown in Figure 1 reflects the discipline specific internal coverage of Table 2. Both are dominated by the relative small School of Physics^24^ followed by further STEM faculties and the School of Business Administration and Economics. In these disciplines, querying OpenAlex is more successful than FREYA and the ORCID public API in case of UOS.

Looking exclusively at the subset of researchers with verified ORCID iDs in Figure 2, the picture shifts, with STEM and SSH subjects moving closer together. While the School of Educational and Cultural Studies achieves a coverage of only 17% in all external data sources, the other SSH subjects reach values between 60 and 75%, and Law even reaches full 100%. In contrast to STEM disciplines these researchers are better found *via* FREYA than OpenAlex. Accordingly, it seems that especially researchers from those disciplines with lower ORCID usage can be found easily in FREYA. One reason could be that FREYA tends to map publications on repositories, while Crossref better reflects the publishing world.

### Limitations

All results are outcome of project TAPIR (TeilAutomatisiertes Persistent-Identifier-basiertes Reporting) carried out by TIB (technical part) and UOS (use cases). This study investigates these two German research institutions. Numbers in general are quite small and limited to full professors and project managers in case of UOS. The currently developed and implemented local CRIS of UOS focuses on these groups of researchers, which means that only this data could be evaluated. This prioritization by UOS management is due to an annual reporting mandate of the Lower Saxony Ministry for Science and Culture (MWK) for projects funded by public or private research sponsors, most notably the German Research Foundation (DFG), the Federal Ministry of Education and Research (BMBF), and the European Research Council (ERC). In a further development stage, it is planned that UOS CRIS will include early career researchers (e.g., junior professors) and research assistants.

The outcome of the analysis of TIB's ORCID coverage might be biased by TIB's role as scholarly infrastructure provider. It coordinates the ORCID-DE consortium and is generally focused on research on digital libraries and infrastructures which consequences to the institutes profile a higher affinity for using PIDs among their research staff can be assumed.

Conclusions and transfer of the results to other institutions are therefore only possible to a limited extent. In this sense this investigation is a prototypic one. As the scripts of the queries are published open access (Mierz, 2022), institutions of different type, size, scope, and national communities are invited to adopt and reuse them for further investigations.

### Conclusion and outlook

In all cases, querying external data sources for both institutions, the coverage of ORCID iDs is still too low to be used as a reliable and adequate data source for reporting needs on its own. It still requires additional (manual) corrections and validations to enrich external ORCID person lists, to ensure the same quality as provided by internally maintained lists. The current level of coverage of researchers that could automatically be mapped by the institutional identifier ROR is still not sufficient to use persistent identifier-based extraction of research results for (automated) standard reporting, but can be useful to maintain and to enrich local CRIS.

**Figure 3:**
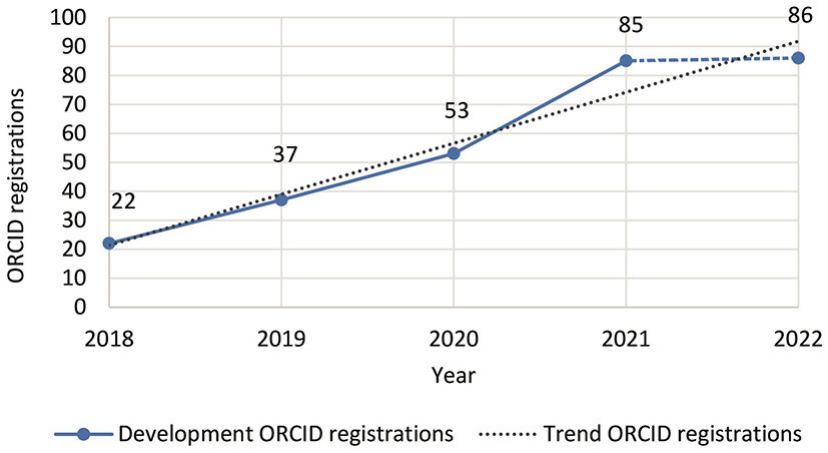
Development of ORCID registrations for TIB employees (source: ORCID Member Portal, March 2022).

**Figure 4:**
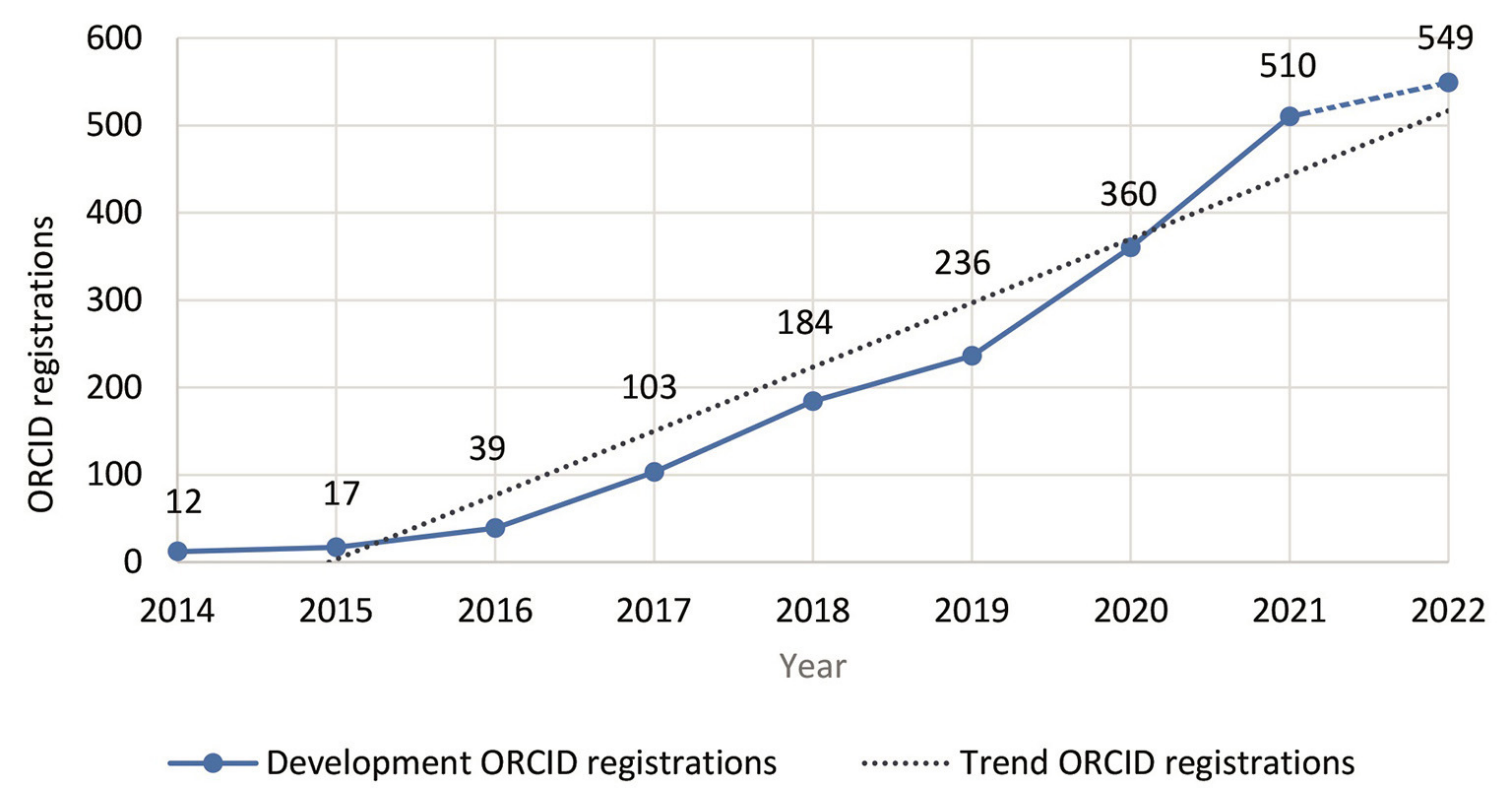
Development of ORCID registrations for UOS employees (source: ORCID Member Portal, July 2022).

ROR as an institutional identifier has just launched in 2019, but promises enormous potential for affiliation based searching. ORCID registrations are also steadily increasing—both worldwide and at the two institutions under consideration, TIB (Figure 3) and UOS (Figure 4).^25^

For semi-automated queries to make a substantial contribution to research reporting, it would be necessary to ensure that PIDs are reliably and consistently used in research and publishing processes.^26^ The new ORCID Affiliation Manager for ORCID consortium members can be an important milestone for this. With this tool, institutions can enter authoritative affiliation statements in ORCID records of their researchers and establish contact with the researchers to inform them about the benefits of using PIDs.^27^

Another important impetus at UOS is the launch of the university's bibliography osnaScholar in June 2022.^28^ When osnaScholar (using OpenAlex as one of its data sources) is fully developed and a workflow is established that includes a correction and completion function to support researchers, they will only have to maintain their publication lists in one place, and will be able to access and reuse these publication lists. The local CRIS integrates osnaScholar data for maintaining researcher profiles. In this context, the benefit for researchers to use PIDs consistently becomes evident. A strong policy supported by the university management (e.g., affiliation policy, Open Science policy etc.), the federal government and funding agencies can promote the internal dissemination of ORCID and the usage of PIDs as well (Mendes Moreira et al., 2015; Porter, 2021). Especially when research funders and the public sector make their own databases of project FAIR (Wilkinson et al., 2016) and thus exemplify open science themselves. The effectiveness of these actions should be accompanied by regular monitoring of internal and external ORCID coverage.

Concerning future development potentials of external data sources newcomer OpenAlex is auspicious and already a valuable resource for research-related investigations. If OpenAlex were to integrate other data sources and types in the future (e.g., the DataCite metadata schema, research data, or funding information), (semi-) automated reporting could make a significant progress.

Further research should address the second stage query already outlined in the introduction—the relation person (ORCID iD) to works (DOI): to what extent can publications and other research outputs be extracted (semi-)automatically from open data sources and fed into institutional systems such as university bibliographies or CRIS? For this purpose, prepared queries (person-works) for additional external data sources (e.g., OpenAIRE Research Graph^29^ and Crossref) are already available for further investigations (Mierz, 2022). Are there discipline specific effects, e.g., because publication types, publications languages or resource types are referenced (or covered) differently in external data sources? How well does a CRIS based on open data sources compared to commercial products in terms of reporting requirements?

The limitations mentioned above must be taken into consideration when transferring the findings of this study to other subjects, countries or types of institutions. Nevertheless, this study can serve us as a starting point for further research. The relationship between academic age, publication history, and discoverability in external data sources deserves further investigation. For this purposes the developed scripts are open access and reusable.

## Data availability statement

The datasets presented in this study can be found in online repositories. The names of the repository/repositories and accession number(s) can be found below: osnaData, https://osnadata.ub.uni-osnabrueck.de/dataverse/tapir; https://doi.org/10.26249/FK2/ZGVJAJ; https://doi.org/10.26249/FK2/ADQVEK.

## Author contributions

SS and CH generated the ideas for this article and the design of the study. SH-W wrote the section-Introduction. CH wrote the section-Related works. SM wrote the section-Selection of open data sources and developed the query tool used for this study. KS gathered data for the study, conducted data comparison, and created figures and tables. WM, SB, and KS wrote sections-Results and discussion and prepared/edited the final submitted version of the manuscript. All authors contributed to the manuscript and final revisions, read, and approved the submitted version.

## Funding

This work was funded by the Federal Ministry of Education and Research (BMBF Grant Funding Identifier 16PH20001A/16PH20001B) and is a result of the research project TAPIR—partially automated persistent identifier based reporting.

## Conflict of interest

The authors declare that the research was conducted in the absence of any commercial or financial relationships that could be construed as a potential conflict of interest.

## Publisher's note

All claims expressed in this article are solely those of the authors and do not necessarily represent those of their affiliated organizations, or those of the publisher, the editors and the reviewers. Any product that may be evaluated in this article, or claim that may be made by its manufacturer, is not guaranteed or endorsed by the publisher.

## Footnotes

1 In the past 20 years, third-party funding revenues in universities and higher education institutions increased by a factor of 3.3 (Statistisches Bundesamt, 2021).

2 https://info.orcid.org/enter-once-reuse-often/ (accessed August 1, 2022).

3 Research Organization Registry, https://ror.org (accessed August 1, 2022).

4 Open Researcher and Contributor ID, https://orcid.org (accessed August 1, 2022).

5 Digital Object Identifier, https://dx.doi.org/ (accessed August 1, 2022).

6 DataCite, https://datacite.org/ (accessed August 1, 2022).

7 For example, to fulfill the FAIR (=“Findable, Accessible, Interoperable, and Re-usable”) principles for research data, e.g., assigning DOIs to research data supports persistent findability, accessibility, interoperability with other systems and contents, and reusability of data and its metadata (Wilkinson et al., 2016).

8 ROR was launched in 2019 with seed data of GRID and synced with GRID until GRID ceased its Service in 2021. The first independent update of the ROR registry was released in March 2022 (Gould, 2022).

9 Crossref, https://crossref.org (accessed August 1, 2022).

10 https://support.datacite.org/v1.3/docs/what-are-the-resource-types-for-datacite-dois (accessed August 1, 2022).

11 See, e.g., PID Graph developed in EU-funded FREYA project (Fenner and Aryani, 2019).

12 https://www.tib.eu/de/publizieren-archivieren/pid-service (accessed August 1, 2022).

13 https://www.orcid-de.org/konsortium (accessed August 1, 2022).

14 This comprises all fundings for research activities by external, national and international financial sources.

15 https://cordis.europa.eu/project/id/777523 (accessed August 1, 2022).

16 E.g., public ORCID data and ROR registry are fully integrated in FREYA (Fenner, 2020).

17 https://api.datacite.org/graphql (accessed August 1, 2022).

18 https://info.orcid.org/what-is-orcid/ (accessed August 1, 2022).

19 https://info.orcid.org/researchers/ (accessed August 1, 2022).

20 https://api.openalex.org (accessed August 1, 2022).

21 The anonymized datasets are published for further investigation (Schnieders, 2022a,b).

22 https://www.uni-osnabrueck.de/en/home/ (accessed August 1, 2022); not including Medicine and Engineering.

23 Just about 9 Mio Crossref publications are indexed in FREYA Fenner (2020).

24 The School of Physics at UOS employs only 4 full professors, while a fully developed physics department at German universities corresponds to an average of 18–22 full professors (Moravetz-Kuhlmann, 2010).

25 Including UOS employees who are not on the internal ORCID person list, which is limited to full professors and project managers.

26 The current development status of an automatically filled CRIS can be traced with the Generate2VIVO import tool developed in the TAPIR project (Technische Informationsbibliothek and Osnabrück University, 2021).

27 The ORCID Member API could be used to query researchers of an institution in more detail, but this study is limited to querying the ORCID public API because it is free to use and the search strategy is thus adaptable.

28 https://osnascholar.ub.uni-osnabrueck.de/ (accessed August 1, 2022).

29 OpenAIRE Research Graph, https://graph.openaire.eu/ (accessed September 8, 2022).
